# Angiofibroma of the Vagina Presenting with Abnormal Vaginal Bleeding: A Case Report from Ethiopia and Review of the Literature

**DOI:** 10.1155/2019/1486387

**Published:** 2019-06-02

**Authors:** Chuchu Arega, Wubshet Girma, Jose Manuel Sanchez Diaz

**Affiliations:** ^1^Department of Obstetrics and Gynecology, Jimma University, Ethiopia; ^2^Department of Obstetrics and Gynecology, Jimma University Medical Center, Ethiopia

## Abstract

**Background:**

Angiofibroma is an uncommon type of vascular benign tumor that is made up of blood vessels and fibrous (connective) tissue. First described in 1997, it usually occurs in middle aged females and is clinically often thought to represent a cyst. Unlike most of the other site specific vulvovaginal mesenchymal lesions, cellular angiofibroma has a marked predilection for the vulva with only occasional examples reported in the vagina.

**Case Detail:**

A 17-year adolescent nulligravid girl presented with a history of irregular vaginal bleeding of two-year duration and history of lower abdominal swelling; on examination, she had pale conjunctiva, 20-week sized firm, irregular, nontender abdominopelvic mass, and a firm huge anterior vaginal wall mass, with difficulty to reach at the cervix and hemoglobin of 9.7 gm/dL, and a diagnosis of cervical myoma plus anemia was made, which was supported by imaging studies. Finally it was found to be angiofibroma of the vagina.

**Conclusion:**

Angiofibromas are benign tumors, which rarely occur in vagina. Although middle aged females are affected more, angiofibromas can affect females of reproductive age group and can cause abnormal uterine bleeding.

## 1. Introduction

Angiofibroma is an uncommon type of vascular benign tumor that is made up of blood vessels and fibrous (connective) tissue. The lesion first recorded by Chelins in 1847 and the name “angiofibroma” was given by Friedberg in 1940. Despite being benign the lesion can present as a local malignancy. Angiofibroma, first described by Nucci in 1997, usually occurs in middle aged females and is clinically often thought to represent a cyst. Since 1997 only few studies have been published in the literature, most of which consisted of single case reports or reviews [1].

Juvenile angiofibroma (JNA) appears almost exclusively in adolescents between 14 and 25 years. In young female population angiofibroma is extremely rare and only about 15 similar cases were identified. According to literature and our own clinical experience the clinician must be aware of the possibility of the occurrence of angiofibroma in unusual sites in prepubertal age. Unlike most of the other site specific vulvovaginal mesenchymal lesions, cellular angiofibroma has a marked predilection for the vulva with only occasional examples reported in the vagina [2].

The pathogenesis of angiofibroma is still unclear but there are several theories regarding the formation of angiofibroma; the most widely accepted is the angiogenic and the histogenetic theory. Few studies have examined the sex hormones (estrogen and progesterone), genetic, and an increased intracellular reactive oxygen species has been proposed [3].

## 2. Case Presentation

A 17-year-old adolescent nulligravid girl whose last menstrual period was four days back referred with the diagnosis of low lying myoma after being transfused with four units of blood. She presented to the Gynecology Outpatient Department on 22 July 2018, with history of irregular vaginal bleeding of 2-year duration.

She gives history of lower abdominal swelling which was small initially and progressively enlarged to attain the current size for the last 13 months. She is sexually active but not married. She has no history of abdominal pain, urinary, or bowel complaint, has no bleeding from other sites, and has no significant past medical and surgical history noted.

On examination, patient was conscious, coherent with blood pressure of 100/60 mmHg, pulse 80 /min, and temperature normal and has pale conjunctiva, cardiovascular, and respiratory systems normal. Abdominal examination revealed 20-week sized nontender, firm, irregular, fixed, and lower border unreachable mass. On vaginal examination, a firm huge nontender growth attached to the anterior vaginal wall. It was difficult to reach at the cervix. A clinical diagnosis of cervical myoma was made.

**Figure 1:**
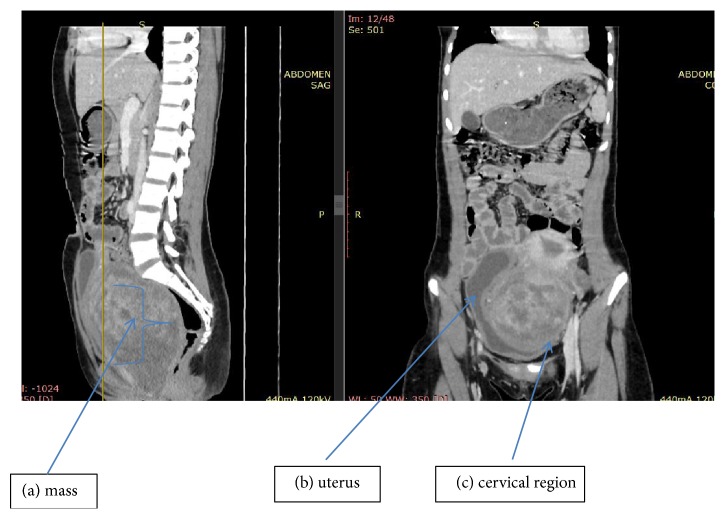
Computed Tomography (CT) scan of the abdomen and pelvis. Arrow heads show hypodense contrast enhancing mass (a) seen on the uterus (b) arising from the cervical region (c).

Her blood investigations on arrival showed hemoglobin of 9.7 gm/dL and were transfused one unite blood and the hemoglobin elevated to 10.7 gm/dL. Ultrasonography showed empty uterus with normal size echotexture and pushed up in to the abdomen. There is 10 by 12 cm hypoechoic mass arising from the cervix filling the vaginal canal with these the diagnosis of cervical myoma was made. The diagnosis necessitated Computed Tomography (CT) scan of the abdomen and pelvis which helped to know the extent of the mass and reported hypodense contrast enhancing mass seen on the uterus arising from the cervical region measures about 10.3 cm by 14.4 cm. The mass grows down in to the vagina and concluded with the diagnosis of contrast enhancing cervical mass likely myoma (Figure 1).

**Figure 2:**
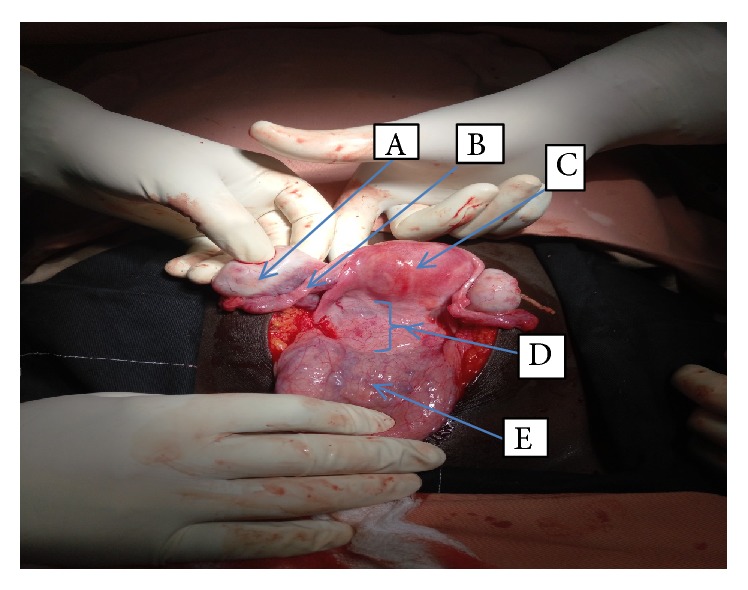
The arrows in the above picture taken intraoperative show the right ovary (A), the right fallopian tube (B), uterus (C), vaginal wall mass pushed up, (D) and bladder (E).

**Figure 3:**
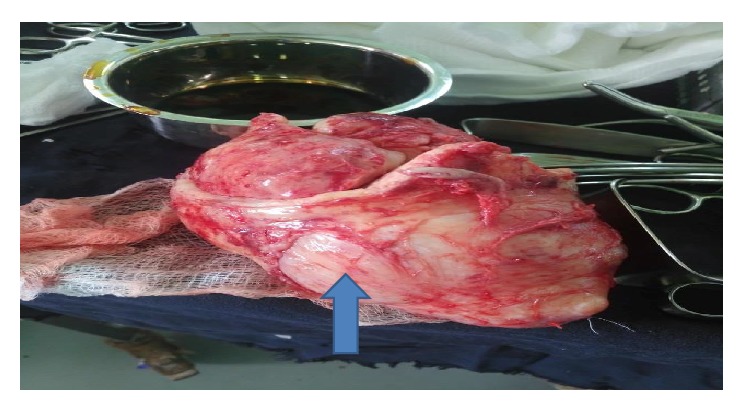
Gross appearance of the excised mass (blue arrow).

**Figure 4:**
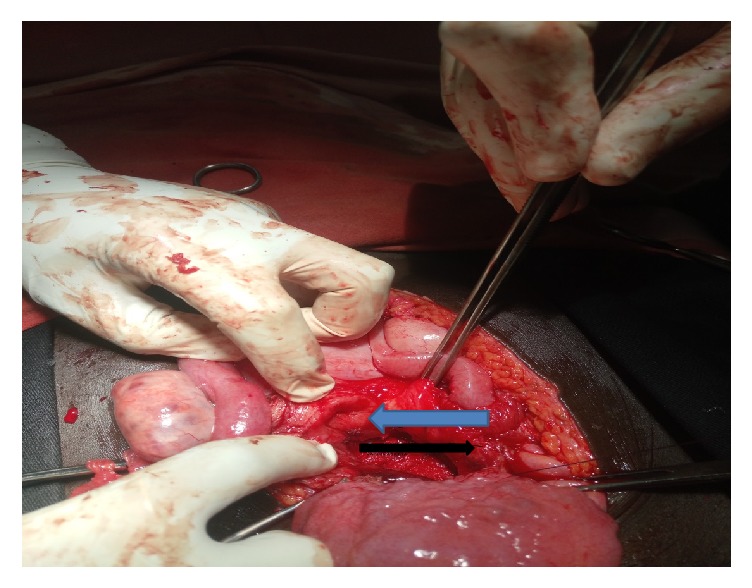
The gross picture of the cervix (blue arrow) and anterior vagina (black arrow) after excision of the mass.

Understanding the complexity of her surgery and possible postoperative morbidity and mortality, surgical, anesthesiology, nursing, and recovery room teams was assembled with gynecologic oncology team. She was prepared for elective laparotomy the day before the surgery. On 18 August 2018, the patient was taken to operating room, after the general anesthesia given; she was positioned in a supine position. The abdomen cleaned with povidone iodine and draped with sterile towels and midline vertical incision was made. Intraoperatively, bilateral fallopian tubes and ovaries, the uterus, and urinary bladder grossly look normal; there is 10 cm by 15 cm sized firm mass between the vagina and the lower uterine segment (Figure 2). Vesicouterine peritoneum incised the bladder reflected away from the lower uterine segment and upper vaginal wall, about 4 cm vertical incision was made on proximal anterior vaginal wall, and dissection of the mass away from the anterior vaginal wall was tried but it was difficult to have a clear cleavage line to excise the whole mass. Then another 6 cm longitudinal incision was made on the posterior vaginal wall; sharp and blunt dissection were made to separate the bulk of mass away from the vaginal wall; finally excision of the mass from the base within 1 cm of normal vaginal tissue was performed to carefully remove a 15 × 10 × 7 cm encapsulated mass (Figure 3). The anterior vaginal wall, extension of incision on the cervix, and posterior vaginal wall closed separately with vicryl number 0 in two layers (Figure 4). Hemostasis was secured, correct count was reported, the fascia and skin were closed using delayed absorbable stitch. The surgery was completed after 2hrs and 20 minutes.

**Figure 5:**
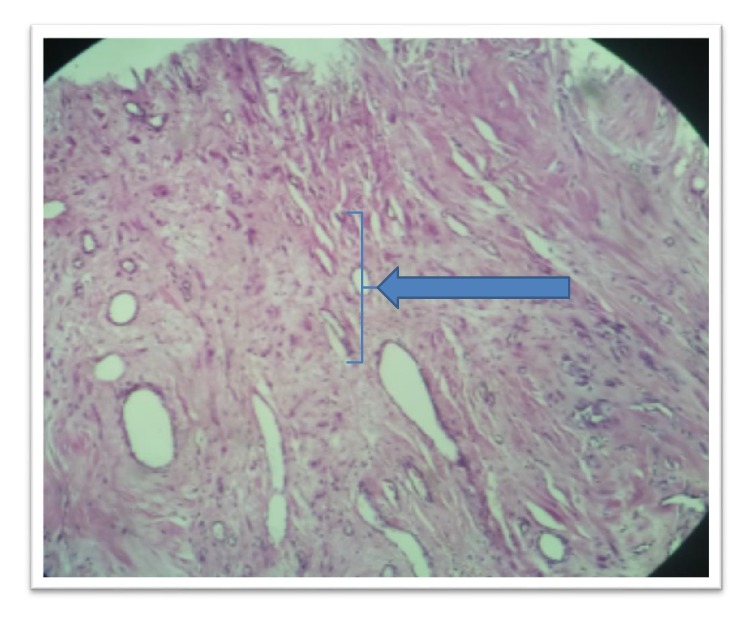
Microsection shows tumor tissue fragments comprised of abundant fibrous tissue (arrow heads) having benign proliferating spindle cells, myxoid areas, and proliferating dilated blood vessels in between.

The excised mass was sent for histopathological examination; the patient recovered completely and discharged on 6th postoperative day. The final pathological diagnosis of the mass was vaginal angiofibroma (Figure 5).

## 3. Discussion

Angiofibroma is a small, well-circumscribed, mostly asymptomatic, typically slow growing, and benign stromal tumor and most often found in the distal genital region. It occurs in both the genders with equal predilection; in women, it is mostly observed in the fifth decade (average age: 46.1 years). In one study 79 affected women have an age at presentation ranging from 20 to 77 years with mean age being 46.1 years [4]. In this case it presented in a 17-year-old nulligravid girl.

The most common anatomic site is the vulvovaginal region; in particular labium major area and vulva overall and vaginal involvements have rarely been reported [5]. In this case, the vagina was involved by this tumor. Clinically, it presents as a bleeding polypoidal or nodular mass [6] and our case presented with abnormal vaginal bleeding and abdominal swelling causing anemia requiring blood transfusion.

Angiofibromas usually grow insidiously and reach a huge size before they are detected. Their largest size has ranged from 3.8 cm to 25 cm [7]. In this case, the size was 14cm. The most common clinical diagnoses before surgery were a Bartholin's cyst in 48% of cases, a not-specific solid mass in 28 % of cases, vulvar cyst in 12 % of cases, leiomyoma in 8 % of cases, and lipoma 4 % of cases [8]. In our case it was considered as cervical myoma.

## 4. Pathological Features

A macroscopic/microscopic description was reported in most of angiofibroma. Grossly is described as white or yellowish nodules, mostly firm and partly gelatinous of cystic in appearance, with a cut surface of white tan to grayish in color [9]. In this case, gross examination shows one tumor mass incompletely sectioned triangular well circumscribed measuring 14 x10x6.5 cm solid smooth surface with fibrous bands some stitch and congestion with focal hemorrhage. Cut surface; hard consistency of whorly surface homogenous yellow white with focal areas of nodularity and myxoid.

Microscopically, angiofibroma is a cellular neoplasm, composed of bland spindle shaped cells, proliferating in an edematous to fibrous stroma, containing wispy collagen bundles, numerous small to medium-sized thick-walled, most of the time hyalinized, vessels, and a small component of adipose tissue [10]. In this case, microsection shows tumor tissue fragments comprised of abundant fibrous tissue having benign proliferating spindle cells myxoid areas and proliferating dilated blood vessels in between.

In most of the analyzed cases, treatments with surgical approach consist of a simple local excision or a “shelling out” seems to be adequate [11]. In this case she was treated with surgical excision of the mass.

## 5. Conclusions

Angiofibroma in women represents a distinct benign neoplasm with a broad anatomic distribution even if it is mainly localized in the vulvovaginal area. This lesion may exhibit some variations in its phenotypic features. Treatment of simple local excision or a “shelling out” of the lesion appears to be adequate and effective to avoid anemia from abnormal vaginal bleeding, recurrences, and injuries to surrounding tissues.

## Abbreviations

JNA: Juvenile angiofibroma
CT: Computed Tomography
ROS: Reactive oxygen species.
